# The Detection of Circulating Antigen Glutathione S-Transferase in Sheep Infected with *Fasciola hepatica* with Double-Antibody Sandwich Signal Amplification Enzyme-Linked Immunosorbent Assay

**DOI:** 10.3390/ani14030506

**Published:** 2024-02-03

**Authors:** Jiahui Duan, Nan Zhang, Shaoxiong Liu, Jianhua Li, Pengtao Gong, Xiaocen Wang, Xin Li, Xu Zhang, Bo Tang, Xichen Zhang

**Affiliations:** State Key Laboratory for Diagnosis and Treatment of Severe Zoonotic Infectious Diseases, Key Laboratory for Zoonosis Research of the Ministry of Education, Institute of Zoonosis, College of Veterinary Medicine, Jilin University, Changchun 130062, China; duanjh1001@163.com (J.D.); n_zhang@jlu.edu.cn (N.Z.); jlulixin0928@163.com (X.L.);

**Keywords:** *F. hepatica*, circulating antigen, double-antibody sandwich SA-ELISA, glutathione s-transferase

## Abstract

**Simple Summary:**

As a global zoonotic parasitic disease, fasciolosis can cause serious economic losses to animal husbandry. The timely detection of *Fasciola hepatica* (*F. hepatica*)-infected animals and the adoption of epidemic prevention measures are of great significance. This manuscript provides a new technical method, a biotin–streptavidin signal amplification ELISA (SA-ELISA), based on anti-rFhGST monoclonal and polyclonal antibodies, which can detect circulating antigens in the serum of sheep infected with *F. hepatica* and achieve early detection. This study explores its application value in immune diagnosis, laying the foundation for the development of serum detection preparations for *F. hepatica* infection.

**Abstract:**

Fasciolosis is a global zoonotic parasitic disease caused by *F. hepatica* infection that is particularly harmful to cattle and sheep. A biotin–streptavidin signal amplification ELISA (streptavidin-ELISA/SA-ELISA) based on circulating antigens can allow for the early detection of *F. hepatica*-infected animals and is suitable for batch detection. It is considered to be a better means of detecting *F. hepatica* infection than traditional detection methods. In this study, using the serum of sheep artificially infected with *F. hepatica*, the cDNA expression library of *F. hepatica* was screened, 17 immunodominant antigen genes of *F. hepatica* were obtained, and glutathione s-transferase (GST) was selected as the candidate detection antigen. Firstly, the GST cDNA sequence was amplified from *F. hepatica*, followed by the preparation of recombinant protein GST (rFhGST). Then, monoclonal and polyclonal antibodies against rFhGST were prepared using the GST protein. Afterward, the immunolocalization of the target protein in the worm was observed via confocal microscopy, and it was found that the GST protein was localized in the uterus, intestinal tract, and body surface of *F. hepatica*. Finally, a double-antibody sandwich SA-ELISA based on the detection of circulating antigens was established. There was no cross-reaction with positive sera infected with *Dicrocoelium lanceatum* (*D. lanceatum*), *Haemonchus contortus* (*H. contortus*), *Neospora caninum* (*N. caninum*), or *Schistosoma japonicum* (*S. japonicum*). Forty serum and fecal samples from the same batch of sheep in Nong’an County, Changchun City, Jilin Province, China were analyzed using the established detection method and fecal detection method. The positive rate of the SA-ELISA was 17.5%, and the positive rate of the fecal detection method was 15%. The detection results of this method were 100% consistent with commercial ELISA kits. A total of 152 sheep serum samples were tested in Nong’an County, Changchun City, Jilin Province, and the positive rate was 5.92%. This study laid the foundation for the development of serological detection preparations for *F. hepatica* infection based on the detection of circulating antigens.

## 1. Introduction

Fasciolosis is a zoonotic parasitic disease caused by *Fasciola spp*. parasitizing the liver and bile ducts of animals and humans. It is distributed worldwide and is common in ruminants such as cattle and sheep. The causative pathogens include *F. hepatica*, *Fasciola gigantica* (*F. gigantica*), and *Fasciola intermedia (F. intermedia*) [1,2,3,4,5]. More than 700 million domestic animals are infected with *F. hepatica* worldwide, and the economic loss it causes to animal husbandry is more than USD 3.2 billion every year, seriously affecting the development of animal husbandry [6,7,8]. At present, the control of *F. hepatica* infection mainly relies on chemical drugs, such as triclabendazole, but drug resistance is prone to occur, and there is no effective *F. hepatica* vaccine that has been produced and marketed [9,10]. Therefore, it is of great significance to detect *F. hepatica*-infected animals in the early stages of infection and take preventive and control measures before the onset of the disease. Compared with fecal detection and molecular biology detection, immunological detection has certain advantages due to its high sensitivity and suitability for batch detection [11,12,13]. The basis of immunological detection is the detection of antigens or antibodies. After infection with *F. hepatica*, the specific antibodies produced by the host will persist in the body for more than 1 month [14,15]. However, various antigens secreted or excreted by *F. hepatica* exist in the host’s circulatory system and can be metabolized by the host in a short time. Their existence reflects the current parasite infection status of the hosts [16,17]. Therefore, the detection of *F. hepatica* infection based on circulating antigens has certain diagnostic advantages. The reported antigens for the detection of *F. hepatica* mainly include cathepsin L (CatL), saposin-like protein (SAP), and fatty acid-binding protein (FABP) [18,19,20,21], but in general, there are relatively few antigens to choose from. There have been related studies based on the detection of circulating antigens in the case of *F. gigantica* infection; however, there are few reports on the detection of circulating antigens in *F. hepatica* infection [22,23]. Further research is needed. Therefore, this study established a double-antibody sandwich SA-ELISA for *F. hepatica* infection by using circulating antigens and explored its application value in immunodiagnosis, laying the foundation for the development of serological detection preparations for *F. hepatica* infection.

## 2. Materials and Methods

### 2.1. Serum and Fecal Samples

Positive and negative serum samples for *F. hepatica* infection, positive serum samples for *D. lanceatum* infection, positive serum samples for *H. contortus* infection, and positive serum samples for *N. caninum* infection in sheep were preserved by the Key Laboratory of Zoonosis Research, Ministry of Education, Institute of Zoonosis, College of Veterinary Medicine, Jilin University. Serum that was positive for *S. japonicum* infection in sheep was donated by Professor Jinming Liu from the Shanghai Veterinary Research Institute, Chinese Academy of Agricultural Sciences. Serum samples from sheep artificially infected with *F. hepatica* and four adult *F. hepaticas* samples were donated by Professor Chunren Wang of Heilongjiang Bayi Agricultural University in China. Forty serum samples and forty fecal samples (serum and fecal samples were from the same batch of sheep, one-to-one correspondence between fecal and serum samples) were collected from Nong’an County, Changchun City, Jilin Province, China. Furthermore, 152 serum samples of sheep to be tested were collected from Nong’an County, Changchun City, Jilin Province, China.

### 2.2. Screening of Candidate Detection Antigen Genes of F. hepatica

The X-blue bacteria and the phage containing the cDNA expression library (the cDNA expression library was prepared by Takara Company (Beijing, China using *Fasciola hepatica* cDNA; the storage capacity was 2 × 10^7^ pfu/800 μL, and the prepared library was stored in our laboratory) of *F. hepatica* were cocultured in NZY agar plates [24]. Protein expression was induced by using isopropyl β-D-thiogalactoside (IPTG: 0.1 mM), and the protein was transferred to the NC membrane (Bio-Rad Laboratories, Inc., Beijing, China) and incubated with serum artificially infected with *F. hepatica*. Phage plaques with obvious protein expression were picked for PCR amplification. The pre-mixed PCR reaction solution contained 8 μL of ddH_2_O, 10 μL of 2 × Master Mix, 1 μL of upstream primer, and 1 μL of downstream primer. The reaction procedure was as follows: pre-denaturation at 95 °C for 3 min, denaturation at 94 °C for 1 min, annealing at 66 °C for 30 s, extension at 72 °C for 1 min, cycling 28 times, and then extension again at 72 °C for 10 min. The forward primer was 5′ CTCGGGAAGCGCGCCATTGTGTTGGT 3′, and the reverse primer was 5′ ATACGACTCACTATAGGGCGAATTGGCC 3′. Sequencing and screening were used to obtain the candidate detection antigen gene of *F. hepatica*.

### 2.3. Expression, Purification, and Identification of the Recombinant Protein rFhGST

The obtained FhGST gene was selected for prokaryotic expression. The primers were designed using Oligo 7 software (Molecular Biology Insights, Inc., Eagle, Cascade, CO, USA), PCR amplification (forward: 5′ ATGGCTGATATCGGATCCATGCCAGCCAAACTCGGATAC 3′; reverse: 5′ AGCTTGTCGACGGAGCTCAGCCGGTGCAGCGTC 3′) was performed using the cDNA (the chloroform/phenol method was used to extract the total RNA of *F. hepatica*, and the reverse transcription method was used to synthesize the cDNA of *F. hepatica*) of *F. hepatica* as a template, and the amplified product was connected to the expression vector pET-32a (+) (Sangon Biotech Co., Ltd., Shanghai, China). After the identification of double enzyme digestion (restriction enzymes: *BamH1* and *Sac1*), sequencing (Sangon Biotech Co., Ltd.), and sequence analysis, the recombinant plasmid was transformed into *Escherichia coli* BL21 (DE3) (Sangon Biotech Co., Ltd.), and the expression of the recombinant protein was induced with IPTG at a final concentration of 0.1 mM (LB medium, shaking culture at 37 °C for 6 h). The *E. coli* pellet obtained from the 2 L culture was resuspended in 200 mL PBS, the cells were sonicated on ice, and the target protein was purified with (Ni Agarose Resin) Ni agarose gel (Kangwei Century Biotechnology Co., Ltd., Beijing, China) according to the instructions. Finally, the purification of the protein was analyzed using SDS-PAGE and Western blotting (antibody: HRP-conjugated His-Tag Monoclonal antibody; dilution ratio: 1:5000).

### 2.4. Preparation of the Anti-rFhGST Polyclonal Antibody

Five hundred micrograms of the purified recombinant protein rFhGST (at a concentration of 1 mg/mL in PBS) was mixed with an equal amount of Freund’s complete adjuvant (Sigma Aldrich Trading Co., Ltd., Shanghai, China), fully emulsified, and used to immunize 2-month-old New Zealand rabbits. On the 15th and 22nd days, the second and third vaccinations were performed using Freund’s incomplete adjuvant. On the 7th day after the third immunization, blood was collected from the rabbit’s ear vein, and the serum was prepared.

An indirect ELISA was used to measure the antibody titer in the rabbit serum. Firstly, 96-well plates (Jet Biofil Co., Ltd., Guangzhou, China) enzyme-labeled plank polystyrene) were coated with recombinant protein rFhGST at a concentration of 1 μg/100 μL, incubated overnight at 4 °C and then added to immunized and nonimmunized rabbit serum samples (rabbit serum diluted with PBST in a ratio of 1:1000 to 1:512,000), and incubated at 37 °C for 2 h. Subsequently, enzyme-labeled sheep anti-rabbit polyclonal antibody (diluted with PBST in a ratio of 1:5000 and incubated at 37 °C for 1 h), TMB substrate solution (100 μL/well, incubated at room temperature for 15 min), and termination solution (2M H_2_SO_4_, 100μL/well) were added to the 96-well plates in sequence. The 96-well plate was washed with PBST between each step. Finally, the absorbance values were read by using the enzyme-linked instrument (Biotek Co., Ltd., Winooski, VT, USA) at a wavelength of 450 nm. If the serum antibody titer reached a ratio of 1:100,000 or more, blood was drawn from the heart, and a large amount of serum was collected. The experimental rabbit was euthanized after blood collection.

The polyclonal antibody was crudely purified via the saturated ammonium sulfate method. In this study, 20 mL of serum was added to 20 mL of PBS solution, and then saturated ammonium sulfate solution was slowly added to form 20%, 50%, and 33% ammonium sulfate solutions in sequence. Afterward, impurities were gradually removed via the centrifugation of the supernatant or sediment. Finally, PBS was used to dissolve the precipitate, and the solution was then placed in a dialysis bag. The solution was dialyzed at 4 °C for 24 h, and the precipitate was removed via centrifugation to obtain the crude IgG supernatant. Afterward, the polyclonal antibody was finely purified using protein A/G mixed column material purification resin (Sangon Co., Ltd., Shanghai, China). SDS—PAGE was used to analyze antibody purification, and Western blotting was used to observe the reactogenicity of the rabbit polyclonal antibody (dilution ratio of rabbit anti-rFhGST polyclonal antibody: 1:10,000).

### 2.5. Immunolocalization of FhGST in F. hepatica

Adult worms of *F. hepatica* were fixed with 10% formalin, washed thoroughly with distilled water, and permeabilized with 3% Triton X-100 for 3 h. The parasites were incubated at room temperature for 10 min with proteinase K at a final concentration of 20 μg/mL (solvent: 0.01M PBS). After blocking with 5% BSA overnight, the rabbit anti-rFhGST polyclonal antibody was used as the primary antibody (dilution ratio: 1:50), and the unimmunized rabbit serum sample was used as the negative control (dilution ratio: 1:50) and incubated at 4 °C for 48 h. Alexa Fluor Cy3 goat anti-rabbit IgG (H + L) (dilution ratio: 1:200) was used as the secondary antibody and was incubated at 4 °C for 24 h. DAPI (Beyotime Co., Ltd., Shanghai, China) was used to stain the nuclei of parasites and was incubated at room temperature for 10 min, and the parasites were observed under a confocal laser microscope.

### 2.6. Preparation of the Anti-rFhGST Monoclonal Antibody

For the preparations, 60 micrograms of the purified recombinant protein rFhGST (1 mg/mL, using PBS as solvent) was mixed with an equal amount of Freund’s complete adjuvant, fully emulsified, and used for initial immunization in BALB/c mice. Then, 30 micrograms of protein was mixed with an equal amount of Freund’s incomplete adjuvant every 14 days for 2–4 immunizations. Mice with high serum antibody titers were selected for splenocyte and thymocyte collection. Then, 50% polyethylene glycol 4000 was used to fuse mouse splenocytes with SP2/0 cells (ATCC Co., Ltd., Gaithersburg, MD, USA). The fused cells were first screened and cultured with a semisolid medium, and hybridoma cell lines were screened for the second and third times by using indirect ELISA. The culture medium of positive hybridoma cells with a high antibody titer that could produce anti-rFhGST antibody was collected, and the SBA Clonotyping™ System/HRP (Southern Biotech, Beijing, China) kit was used to determine the immunoglobulin subclass. After preliminary identification, monoclonal antibodies were produced in the form of ascitic fluid and purified using protein A/G affinity chromatography resin. The concentration of monoclonal antibodies was determined using a BCA protein analysis kit (Thermo Fisher Scientific Co., Ltd., Shanghai, China), and the titer of monoclonal antibodies was measured via an indirect ELISA. A fitting reaction curve model was formed, and the affinity constant was calculated (affinity constant ≈ (150,000 × A)/antibody concentration; A represents the antibody dilution corresponding to the 1/2 OD value on the upper platform) [25]. The purity of the monoclonal antibody was determined via SDS—PAGE, and the reactogenicity of the mouse monoclonal antibody was observed using Western blotting (dilution ratio of anti-rFhGST monoclonal antibody: 1:10,000).

### 2.7. Preparation and Optimization of Biotin-Labeled Rabbit Anti-rFhGST Polyclonal Antibody

Two milligrams of biotin was weighed and dissolved in 590 μL of dimethyl sulfoxide to form a 10 mM biotin solution. An appropriate amount of biotin solution was weighed and added to the purified rabbit anti-FhGST polyclonal antibody solution so that the molar ratios of polyclonal antibody to N-hydroxysuccinimidobiotin (BNHS) were 1:20, 1:40, 1:80, and 1:160. A sandwich ELISA was applied to determine the optimal molar feed ratio between rabbit polyclonal antibodies and BNHS. Firstly, the monoclonal antibody was coated on a 96-well plate at a concentration of 0.375 μg/100 mL (100 μL/well) and was incubated at 4 °C for 10 h. Then, 5% skimmed milk powder was added as a blocking solution (100 μL/well) to the 96-well plate and incubated at 37 °C for 2 h. Then, the positive control serum sample and negative control serum sample were added to the 96-well plate (100 μL/well, diluted with PBST in a ratio of 1:4) and incubated at 37 °C for 2 h. Subsequently, biotin-labeled rabbit anti-rFhGST polyclonal antibody (diluted with PBST in a ratio of 1:2000), HRP-conjugated Streptavidin (diluted with PBST in a ratio of 1:5000), TMB substrate solution (100 μL/well), and termination solution (2M H_2_SO_4_, 100 μL/well) were added to the 96-well plate in sequence. The 96-well plate was washed with PBST between each step. Finally, the results were read using the enzyme-linked instrument. The sandwich ELISA for subsequent experiments was also carried out according to this step.

### 2.8. Establishment of the SA-ELISA

Using the prepared mouse anti-rFhGST monoclonal antibody as the capture antibody and the rabbit anti-rFhGST polyclonal antibody as the detection antibody, a double-antibody sandwich SA-ELISA for detecting FhGST was established. Various experimental conditions were optimized to improve the performance of the SA-ELISA. During the process of optimizing the conditions, uninfected sheep serum was used with added recombinant protein (0.1 μg rFhGST/25 μL serum) as the positive control sample, and uninfected sheep serum without added recombinant protein was used as the negative control sample [26]. First, the mouse anti-rFhGST monoclonal antibody (3, 1.5, 0.75, 0.375, 0.187, 0.094, and 0.047 μg/100 μL in coating buffer on a 96-well plate) was coated in a gradient dilution, and the optimal coating concentration was determined. Gradually diluted rabbit anti-rFhGST polyclonal antibodies (1:100, 1:200, 1:400, 1:800, 1:1600, and 1:3200) were incubated to determine the optimal dilution. The optimal condition was determined when the ratio (positive control/negative control, P/N) of the OD_450nm_ values was the largest. Second, using the determined mouse anti-rFhGST monoclonal antibody coating concentration and rabbit anti-rFhGST polyclonal antibody dilution, blocking was performed with 1% BSA, 5% skimmed milk powder, and 1% gelatine to determine the best type of blocking solution. An equal amount of TMB substrate solution (100 μL) was added dropwise to the 96-well plate and incubated for 5 min, 10 min, 15 min, and 20 min to determine the optimal reaction time of the substrate solution. Third, 20 negative serum samples were tested according to the method established above, and the average value (X) and standard deviation (SD) of the OD_450nm_ values of the samples were calculated. Negative and positive critical values = negative sample OD_450nm_ mean (X) + 3 × standard deviation (SD).

### 2.9. Performance Test of the SA-ELISA

Under the optimized reaction conditions in Section 2.7 and Section 2.8, the infected and uninfected *F. hepatica* sheep serum was diluted according to the ratio of 1:2–1:64 to determine the lowest detectable serum dilution. The established SA-ELISA, the positive and negative serum of *F. hepatica* infection, the positive serum of *N. caninum* infection, the positive serum of *S. japonicum* infection, the positive serum of *H. contortus* infection, and the positive serum of *D. lanceatum* infection were tested to analyze whether there was a cross-reaction. Using the established detection method, the same batch and three different batches of coated ELISA plates were used to analyze 12 serum samples, and the repeatability of the method was evaluated.

### 2.10. Comparison with Fecal Test

Forty samples of feces and serum were collected from the same batch of naturally infected sheep, and the fecal samples were processed using natural sedimentation methods with water washing. After settling the eggs of *F. hepatica* using natural sedimentation with water washing, the fecal sediments were extracted onto a slide, and the eggs of *F. hepatica* were observed under a microscope in the feces to evaluate the infection of *F. hepatica* in sheep. The established SA-ELISA was used to analyze the sheep serum samples, and the detection results of the two methods were compared. Fecal and serum samples corresponded one-to-one. The sheep samples were collected from small farmers in a multi-pond area in November 2020, where there have been previous cases of *F. hepatica* infection.

### 2.11. Comparison with Commercial Kits

Using the 40 sheep serum samples in Section 2.10, a commercial ELISA kit (Jiangsu Jingmei Biotechnology Co., Ltd., Jiangsu, China), *Fasciola hepatica* (FH) ELISA Kit instruction, antigen) and the SA-ELISA established in this study were used for detection, and the detection results of the two methods were compared.

### 2.12. Application of the SA-ELISA

Then, 152 sheep serum samples from Nong’an County, Changchun City, Jilin Province, China were analyzed using the established SA-ELISA method, collected in November 2020, and the infection status of *F. hepatica* in the area was analyzed.

### 2.13. Statistical Analysis

SPSS 19.0 software was used to calculate the mean and standard deviation. GraphPad Prism 5.0 software was used to form charts.

## 3. Results

### 3.1. Screening of Immunodominant Antigen Genes of F. hepatica

**Figure 1:**
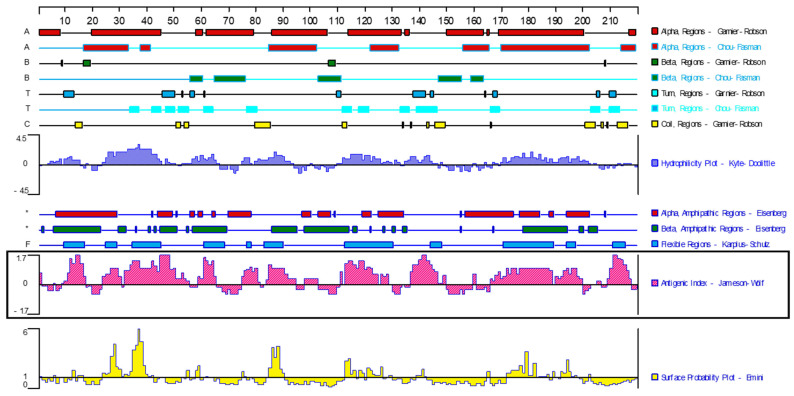
Analysis of antigenic epitopes of GST protein in *F. hepatica*. (Bioinformatics software: DNA Star 7.1; the ID of GST protein: PDB: 2WRT_A; GenBank: EU853672.1).

After culture, evenly spread phage plaques appeared on the NZY agar plate phage medium. After immune screening, 17 immunodominant antigen genes were obtained via PCR amplification and sequencing analysis (Table 1). According to the results obtained with the bioinformatics software of DNAStar 7.1, the GST protein (chain A, class-Mu, PDB: 2WRT_A, GenBank: EU853672.1) contains nine obvious B-cell epitopes (Figure 1). Moreover, GST protein is widely distributed in the tissues of *F. hepatica*, participates in the metabolism of the host [27,28,29,30], and has the potential to be a candidate diagnostic antigen of *F. hepatica*.

### 3.2. Expression, Purification, and Identification of the Recombinant Protein rFhGST

**Figure 2:**
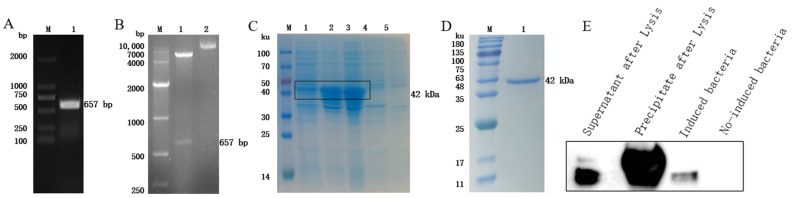
Expression, identification, and purification of rFhGST. (**A**) RT—PCR to amplify the full-length FhGST gene. M: DL2000 DNA marker; 1: full-length FhGST gene. (**B**) Identification of the expression vector via double enzyme digestion. M: DL10000 DNA marker; 1: double digestion product of pET-32a-FhGST; 2: undigested pET-32a-FhGST. (**C**) Identification of the expression of rFhGST via SDS—PAGE and Coomassie brilliant blue staining. M: protein marker; 1: supernatant after lysis; 2: precipitate after lysis; 3: pET-32a-FhGST induced; 4: pET-32a-FhGST not induced; 5: pET-32a induced. (**D**) Identification of the purification of rFhGST by SDS—PAGE and Coomassie brilliant blue staining. M: protein marker; 1: rFhGST after purification. (**E**) Western blot to identify the expression of rFhGST.

The GST gene was amplified via PCR using *F. hepatica* cDNA as a template, and agarose gel electrophoresis analysis revealed an obvious band at the expected position of 657 bp (Figure 2A). The PCR product was recovered, connected to the expression vector, and identified using double enzyme digestion, and the target band was successfully obtained (Figure 2B). After sequencing analysis, the nucleotide sequence was 100% consistent with the sequence in NCBI, and the recombinant expression vector was successfully constructed. IPTG was used to induce the expression of the recombinant protein, and the results of SDS-PAGE showed that the size of the target band was roughly consistent with the expectation, approximately 42 kDa; the recombinant protein was expressed in both the supernatant and the precipitate (Figure 2C). After purification, the recombinant protein had a single band and high purity (Figure 2D). Using the HIS tag antibody to carry out a Western blot analysis, an obvious signal was obtained at the target position, indicating that the recombinant protein rFhGST was successfully obtained (Figure 2E).

### 3.3. Preparation of the Anti-rFhGST Polyclonal Antibody

**Figure 3:**
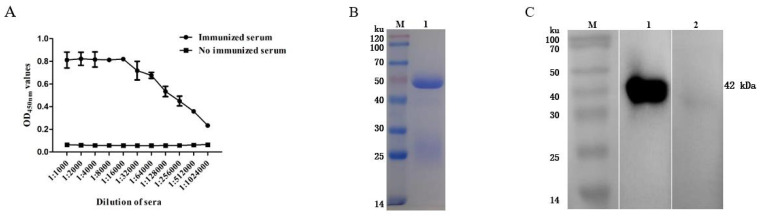
Preparation of rabbit anti-rFhGST polyclonal antibody. (**A**) Anti-rFhGST serum antibody titers. (**B**) Identifying the purification of rabbit anti-rFhGST polyclonal antibody via SDS—PAGE and Coomassie brilliant blue staining. M: protein marker; 1: rabbit anti-rFhGST polyclonal antibody. (**C**) Identifying the reaction of rabbit anti-rFhGST polyclonal antibody to rFhGST via Western blotting. M: protein marker; 1: detection of rFhGST using rabbit polyclonal antibody; 2: detection of rFhGST using nonimmunized rabbit serum.

The antibody titer of the rabbit serum was detected via an indirect ELISA, and the results showed that the antibody titer reached 1:1,024,000 (Figure 3A). The purified rabbit anti-rFhGST polyclonal antibody was subjected to SDS—PAGE and a staining analysis, and an obvious light chain and heavy chain bands could be seen (Figure 3B). After identification and analysis using Western blotting, the prepared polyclonal antibody recognized the recombinant protein rFhGST, indicating that the recombinant protein has good immunogenicity (Figure 3C).

### 3.4. Immunolocalization of FhGST in F. hepatica

**Figure 4:**
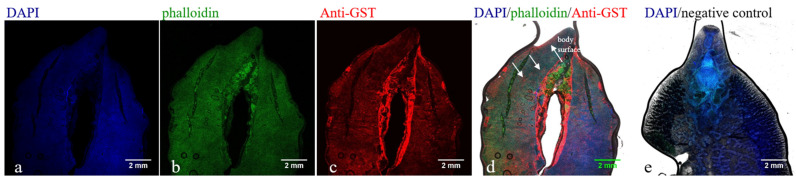
Immunofluorescence staining to identify the reaction of rabbit anti-rFhGST polyclonal antibody to GST protein of *F. hepatica*. (**a**) DAPI marks cell nuclei (blue). (**b**) Actin markers phalloidin marks cytoskeletal membrane components and muscle layer (green). (**c**) Rabbit anti-rFhGST polyclonal antibody marks GST protein of *F. hepatica* (red). (**d**) Merge of figure a, b and c. (**e**) Unimmunized rabbit serum is used as a negative control.

The laser confocal results showed that the fluorescent signals of the *F. hepatica* uterus, intestinal tract, and body surface were stronger, higher than those of other parts, and significantly different from the negative control (Figure 4). The GST protein of *F. hepatica* exists in the uterus, intestinal tract, and body surface of *F. hepatica*. The prepared antibody can recognize parasite proteins and has good reactogenicity.

### 3.5. Preparation of the Mouse Anti-rFhGST Monoclonal Antibody

**Figure 5:**
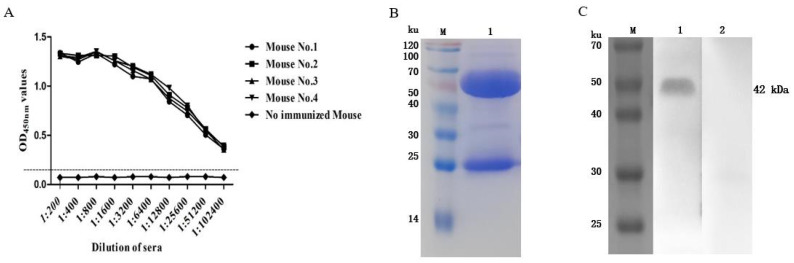
Preparation of mouse anti-rFhGST monoclonal antibody. (**A**) Titers of anti-rFhGST antibodies in mouse sera after immunization. (**B**) Identifying the purified mouse anti-rFhGST monoclonal antibody 2D3 via SDS—PAGE and Coomassie brilliant blue staining. M: protein marker; 1: mouse anti-rFhGST monoclonal antibody. (**C**) Identifying the reaction of mouse anti-rFhGST monoclonal antibody with rFhGST via Western blotting. M: protein marker; 1: detection of rFhGST using mouse monoclonal antibody; 2: detection of rFhGST using nonimmunized mouse serum.

After immunization, the serum antibody titers of the four mice all exceeded 1:102,400 (Figure 5A), and the next step of the cell fusion test could be carried out. After screening and fusion, five positive hybridoma cell lines that can produce higher concentrations of antibodies were obtained, numbered 2D3, 3D8, 3E5, 4E3, and 6F3. Among them, 2D3 is the IgG2b heavy chain subtype, the others are classified as the IgG1 heavy chain subtype, and all light chains are classified as the kappa isotype. Among them, the monoclonal antibody 2D3 has the highest affinity constant and the strongest affinity, so the 2D3 antibody was used for subsequent experiments (Table 2). The purified mouse anti-rFhGST monoclonal antibody 2D3, analyzed using SDS—PAGE, showed clear immunoglobulin heavy chain and light chain bands, and the obtained monoclonal antibody was of high purity (Figure 5B). The monoclonal antibody 2D3 was used as the primary antibody to carry out Western blotting, and it was found that the monoclonal antibody could react with the recombinant protein, but the negative control did not react, and the specificity was good. Therefore, the antibody 2D3 was selected as the capture antibody for the detection method of *F. hepatica* infection (Figure 5C).

### 3.6. Optimization of the Molar Feed Ratio of the Rabbit Anti-rFhGST Polyclonal Antibody to BNHS

**Figure 6:**
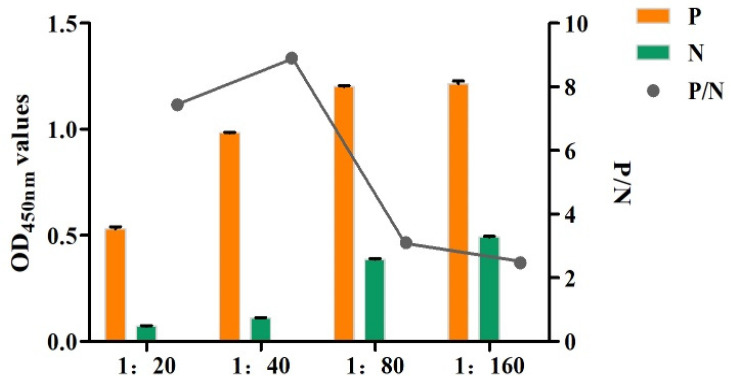
Optimization of the molar feed ratio of the monoclonal antibody to BNHS (1:20–1:160: the molar ratios of polyclonal antibody to N-hydroxysuccinimidobiotin (BNHS) are 1:20, 1:40, 1:80, and 1:160).

The ELISA results showed that when the feed ratio was 1:40, the P/N value was the highest, which was the optimal reaction condition (Figure 6). Therefore, the biotinylated rabbit anti-rFhGST protein polyclonal antibody with a feeding ratio of 1:40 was used as the detection antibody of the SA-ELISA for the subsequent experiments.

### 3.7. Establishment and Optimization of the SA-ELISA

When the coating amount of monoclonal antibody was 0.375 μg/100uL, the OD_450nm_ value of the positive sample still maintained a high level, and the P/N value was high, so it was determined that 0.375 μg/100 uL was the most appropriate coating concentration. Similarly, when the dilution factor of the polyclonal antibody was 1:1600, the blocking solution was 5% skimmed milk powder, the reaction time of the TMB substrate solution was 10 min, and the P/N value was the highest, so it was determined to be the optimal reaction condition.

The results of the critical value test showed that the average (X) of OD_450nm_ of the total samples was 0.095, and the standard deviation (SD) was 0.006. Its positive and negative critical value was 0.114 (X + 3SD). To avoid false positive results, one standard deviation was added or subtracted from the critical value as a suspicious interval. Therefore, when OD_450nm_ < 0.108, it was judged as being negative; when OD_450nm_ ≥ 0.120, it was judged as being positive.

### 3.8. Performance Test of the SA-ELISA

**Figure 7:**
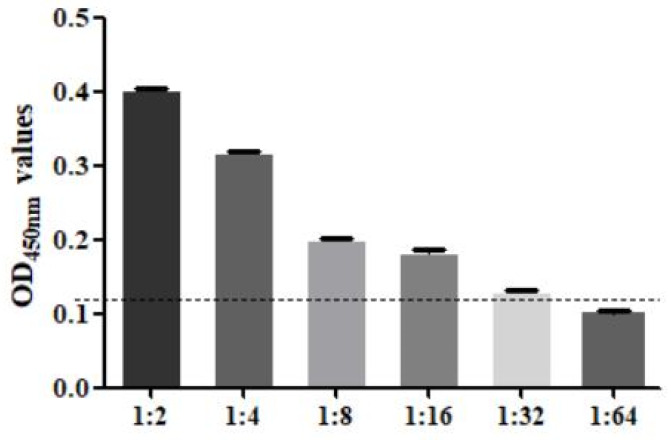
Serum dilution analysis of SA-ELISA (1:2–1:64: the serum in sheep infected *with F. hepatica* was diluted according to the ratio of 1:2–1:64).

**Figure 8:**
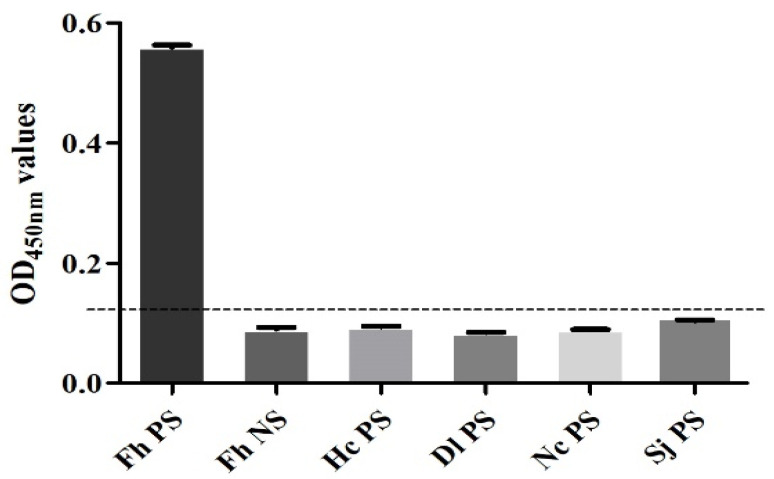
Specificity analysis of SA-ELISA. (Fh PS: positive control serum for *F. hepatica* infection in sheep; Fh NS: negative control serum for *F. hepatica* infection in sheep; Hc PS: positive serum for *H. contortus* infection in sheep; Dl PS: positive serum for *D. lanceatum* infection in sheep; Nc PS: positive serum for *N. caninum* infection in sheep; Sj PS: positive serum for *S. japonicum* infection in sheep).

The sensitivity test found that when *F. hepatica*-infected serum was diluted to 1:32, the result was still positive, so the minimum serum dilution of this method was 1:32 (Figure 7). The specificity test found that only the *F. hepatica*-infected positive control serum samples were positive, and the rest were negative, indicating that the established SA-ELISA had good specificity (Figure 8). The repeatability test results showed that the intra-assay and interassay coefficients of variation in the SA-ELISA were both less than 6% (Table 3).

### 3.9. Comparison with Fecal Test

The results showed that, among the 40 fecal and serum samples, *F. hepatica* eggs were detected in 6 fecal samples, and the positive rate of the stool test was 15%; the circulating antigens of *F. hepatica* were detected in 7 serum samples, and the positive rate of the SA-ELISA was 17.5%. The positive rate of the SA-ELISA was higher than that of the stool test. The fecal test samples and ELISA samples corresponded one-to-one.

### 3.10. Comparison with Commercial Kit Test Results

After testing, 7 of the 40 serum samples tested positive by using the commercial kit and the SA-ELISA established in this study. The results of the SA-ELISA established in this study were consistent with the detection results of the *Fasciola hepatica* (FH) ELISA Kit instruction. The samples of the two detection methods corresponded one-to-one.

### 3.11. Application of the SA-ELISA

Using the established SA-ELISA to detect 152 sheep serum samples from Nong’an County, Changchun City, Jilin Province, China, nine positive samples were obtained with a positive rate of 5.92%.

## 4. Discussion

The commonly used immunological detection method for *F. hepatica* infection is ELISA, the premise of which is to select a suitable antigenic component as a candidate detection antigen [22,31]. The reported secreted or excreted antigen genes of *F. hepatica* include cathepsin L (CatL), saposin-like protein (SAP), and fatty acid-binding protein (FABP) [18,19,20,21]. There have been related studies based on the detection of circulating antigens in *F. gigantica* infection. Anuracpreeda used the sandwich ELISA established with an anti-rFgFABP monoclonal antibody to detect circulating antigens in bovine serum infected with *F. gigantica*. The sensitivity, specificity, and accuracy were 96.7%, 100%, and 99.1%, respectively, showing good results [22,23]. However, there are relatively few studies based on the detection of circulating antigens in the serum of *F. hepatica* infection, and the candidate detection antigen genes need to be further explored [23]. In this experiment, the phage display library was used for antigen screening, which avoided the problem of sourcing the worms and the complicated operation of collecting the secretions and excretions of the worms [32]. After screening, the candidate detection antigen gene GST of *F. hepatica* was identified, and the recombinant protein rFhGST was prepared for subsequent tests.

Glutathione S-transferase (GST) is widely distributed in the tissues of *F. hepatica*, and it is a family of multifunctional enzymes related to cell detoxification, cell repair, and xenobiotic metabolism; its immunodiagnostic application value needs to be further explored [27,28,29,30,33,34]. Previous studies performed immunolocalization on adult worms of *F. hepatica* and found that yolk cells and eggs contained large amounts of GST protein [34]. GST protein staining imprints were also found in the parenchymal cells, outer skin, and intestinal epithelium of *F. hepatica* [28,35,36], indicating that GST protein may exist in the secretion and excretion products of *F. hepatica*. In this study, the antigenic gene FhGST was obtained through immune screening, recombinant protein and polyclonal antibodies were prepared, and the GST protein of *F. hepatica* was immunolocalized. It was found that the protein mainly exists in the uterus, intestinal tract, and body surface of *F. hepatica*. Similar findings were found by LaCourse [35]. Moreover, the GST antigen can be obtained by screening serum infected with *F. hepatica*, indicating that FhGST may be one of the antigen components secreted and excreted by *F. hepatica*, which stimulates the host’s immune response and produces antibodies. At the same time, according to the bioinformatics analysis, it was found that GST protein contains abundant B-cell antigen epitopes and has the potential to become a candidate diagnostic antigen for fascioliasis in sheep. Aguayo V used the GST protein of *F. hepatica* as a serum diagnostic antigen and found that serum antibodies could be detected after *F. hepatica* infection [20]. However, there is no related research on the detection of circulating antigens in the serum of sheep infected with *F. hepatica* based on GST.

In the process of establishing an SA-ELISA based on GST protein for *F. hepatica* infection, recombinant protein was used to prepare monoclonal antibodies, which can obtain antigens with high purity and ensure the screening quality of hybridoma cell lines. By applying the biotin–avidin system to SA-ELISA, since one avidin molecule can label multiple enzyme molecules, it can amplify the reaction signal and improve the sensitivity of the method [23,37]. Javier A Bustos coupled the biotin–avidin system with ELISA to detect serum antigens infected with porcine cysticercosis, showing high sensitivity [38]. The biotin–avidin coupling ELISA established in this study to detect the serum-circulating antigens of *F. hepatica* has a minimum serum dilution of more than 1:32 and has achieved good results. The established SA-ELISA is used to analyze sheep serum samples, has no cross-reaction with positive sera infected with *H. contortus*, *D. lanceatum*, *N. caninum*, or *S. japonicum*, and has good specificity. At the same time, by using the antibody as the key capture reagent of the detection method, the quality of the preparations in different batches is uniform, which ensures the repeatability of the method, and the coefficient of variation within and between batches is below 6%. The same 40 sheep serum samples were detected using the established ELISA and the commercial ELISA kit, and the results were consistent. This result shows that the SA-ELISA established in this study can be used for the detection of *F. hepatica* infection in sheep and has a certain application value. A total of 152 serum samples from Nong’an County, Changchun City, Jilin Province were tested, and the positive rate was 5.92%. This shows that there is *F. hepatica* infection in this area, and it is necessary to strengthen disease prevention and control.

The metacercariae to the larval stage of the parasite is the most pathogenic stage after infecting the host, and it causes the most serious the harm. The early detection of *F. hepatica* infection based on circulating antigens has a higher application value in immunodiagnosis. To determine the early detection value of our established SA-ELISA in *F. hepatica* infection, we used the established SA-ELISA to detect circulating antigens in the serum of three sheep infected with *F. hepatica* on days 1, 3, 13, 21, 36, 89, 94, and 101, which were donated by Professor Chunren Wang of Heilongjiang Bayi Agricultural University in China [39]. We found that circulating antigens in the serums could be detected 13, 21, 36, and 89 days after infection, with the highest serum antigen level being observed at 36 days after infection (Figure S1). Due to the small sample size, the experimental data were not reflected in the Section 2, but they are shown in the Supplementary Materials. The SA-ELISA established in this study can detect the sheep serum circulating antigen GST on the thirteenth day after artificial infection with *F. hepatica*, but no serum-circulating antigen can be detected after the first and third days of infection. This may be because, in the early stages of infection, the amount of antigen secreted is small and difficult to detect. After the 13th day of infection, the secretion of GST protein of *F. hepatica* increased, and it entered the blood circulation system of the host through the dense microvessels in the liver, allowing for early detection [40]. When infected after 89 days, the OD value decreases, which may be due to the role of antibodies in the host body, which form immune complexes with antigens. The amount of free-circulating antigens decreased. Since this study did not obtain sheep serum from days 4 to 12 of infection, whether our method can detect circulating antigens during this period needs further experimental verification. Similarly, Pornanan Kueakhai used the anti-rFgGPx monoclonal antibody to detect circulating antigens in mouse serum 1 week after infection with *F. gigantica*, but he did not conduct artificial infection experiments with *F. gigantica* in large animals such as cattle and sheep [41]. We found that there are almost no reported articles on the detection of circulating antigens in sheep infected with *F. hepatica*, and there is also no commercialized serum circulating antigen detection kit. However, there is an antigen detection kit for detecting *F. hepatica* in feces. Using the MM3-COPRO ELISA kit to detect the infection of *F. hepatica*, *F. hepatica* antigens in the feces of sheep can be detected from the fourth week of infection. The infection detection time of *F. hepatica* infection using this newly established SA-ELISA is 2 weeks earlier than that of the MM3-COPROELISA kit [42]. However, to further validate the practicality of the SA-ELISA test, it is necessary to expand the sample sizes of artificial infections with *F. hepatica* in sheep in the future. When considering the difference in the infection detection time, the ELISA for the detection of blood-circulating antigens showed greater advantages compared with the ELISA for coproantigen detection because the SA-ELISA may be able to achieve early detection and treatment. Meanwhile, the SA-ELISA based on circulating antigen detection can evaluate the therapeutic effects of drugs for fascioliasis [43].

## 5. Conclusions

In summary, this study established a sheep double-antibody sandwich SA-ELISA based on the detection of circulating antigens, which has good sensitivity, specificity, and repeatability. It may be able to achieve the early detection of *F. hepatica* infection and provide a reference for the epidemiological investigation and epidemic prevention of *F. hepatica* infection. This study laid a good foundation for the development of serological detection preparations for *F. hepatica* infection in sheep.

## Figures and Tables

**Table 1:** *F. hepatica* antigen genes obtained via immune screening.

Name	Number	Size/bp
Glutathione S transferase	2WRT_A	657
Vitellin protein B2	THD21181.1	810
Cathepsin L	AAR99519.1	717
Flagellar radial spoke protein 3	TPP67460.1	1320
Hypothetical protein D915_006170	THD22877.1	4380
Hypothetical protein D915_007826	THD21079.1	1383
Hypothetical protein D915_007355	THD21541.1	672
Hypothetical protein D915_010250	THD19012.1	447
Hypothetical protein D915_009223	THD19808.1	375
Hypothetical protein D915_005856	THD23564.1	3357
Hypothetical protein D915_003548	THD25798.1	2520
Hypothetical protein D915_007543	THD21302.1	402
Hypothetical protein D915_009822	THD19402.1	300
Hypothetical protein D915_009429	THD19927.1	7968
Hypothetical protein D915_008999	THD20139.1	1884
Hypothetical protein D915_004302	THD24864.1	489
Hypothetical protein D915_006291	THD22951.1	1086

**Table 2:** Affinity constants of mouse anti-rFhGST monoclonal antibodies.

Number	2D3	3D8	3E5	4E3	6F3
Affinity Constant	1.71 × 10^10^	4.52 × 10^9^	1.06 × 10^10^	1.48 × 10^10^	2.74 × 10^9^

**Table 3:** Intra-assay and interassay coefficients of variation of SA-ELISA.

Sample No.	Intra Batch CV%		Inter Batch CV%	
	$\bar{x}$ ± *s*	CV	$\bar{x}$	CV
1	0.087 ± 0.004	4.14	0.089 ± 0.005	5.29
2	0.085 ± 0.002	2.43	0.09 ± 0.004	3.93
3	0.091 ± 0.004	4.79	0.088 ± 0.005	5.60
4	0.093 ± 0.002	1.86	0.090 ± 0.004	4.70
5	0.095 ± 0.001	1.05	0.090 ± 0.005	5.03
6	0.105 ± 0.003	2.92	0.101 ± 0.006	5.58
7	0.084 ± 0.004	4.76	0.088 ± 0.005	5.65
8	0.085 ± 0.003	3.77	0.086 ± 0.002	2.48
9	0.257 ± 0.003	1.19	0.259 ± 0.005	1.94
10	0.325 ± 0.004	1.28	0.331 ± 0.003	0.85
11	0.194 ± 0.004	1.81	0.199 ± 0.002	1.07
12	0.273 ± 0.002	0.76	0.277 ± 0.003	1.02

## Data Availability

Data are contained within the article and Supplementary Materials.

## Supplementary Materials

The following supporting information can be downloaded at https://www.mdpi.com/article/10.3390/ani14030506/s1, Figure S1: Detection of circulating antigens in serum of sheep artificially infected with *F. hepatica*. Western blot original figures.
