# Supplementary material for: The Detection of Circulating Antigen Glutathione S-Transferase in Sheep Infected with Fasciola hepatica with Double-Antibody Sandwich Signal Amplification Enzyme-Linked Immunosorbent Assay
Source: Animals (Basel). 2024 Feb 3;14(3):506. doi: 10.3390/ani14030506 (PMC10854876; doi:10.3390/ani14030506)
Supplement: Supplementary file 1 [file animals-14-00506-s001.zip › animals-2826471-supplementary.pdf]

Figure S1. Detection of circulating antigen in serum of sheep artificially infected with *F. hepatica*

Through the optimization of conditions in sections 2.7 and 2.8, we obtained the optimal reaction conditions for SA-ELISA and conducted subsequent experiments based on this. The established SA-ELISA was used to analyze the serum samples of 3 sheep at 1 d, 3 d, 13 d, 21 d, 36 d, 89 d, 94 d, and 101 d after artificial infection with *F. hepatica*, which was donated by Professor Chunren Wang. The artificial infection with *F. hepatica* in sheep and a portion of the serum samples collected were indicated in their published article [44]. The main operating method was that the sheep were infected by ingesting lettuce leaves containing *F. hepatica* metacercariae, with approximately 220 metacercariae per sheep. After testing, the sheep serum samples on the 13th, 21st, 36th, and 89th days after artificial infection with *F. hepatica* showed positive results, and the OD value was the highest on the 36th day (Fig S1). Therefore, the time of detectable *F. hepatica* infection was 13 days after infection, and the serum-circulating antigen level was the highest on day 36 after infection.

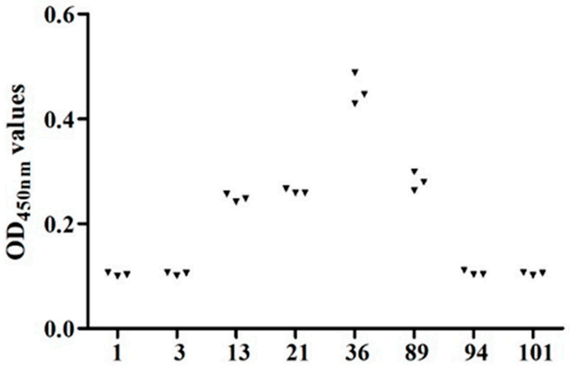

Figure S1. Detection of circulating antigens in serum of sheep artificially infected with *F. hepatica*. (X -axis: number of days infected with *F. hepatica*)

References

44. Lan, Z.; Liu, X.L.; Lv, Q.B.; Zeng, M.H.; Gao, J.F.; Chang, Q.C.; Chen, Y.Y.; Wang, C.R. Proteomic Analysis of Fasciola hepatica Excretory and Secretory Products Co-Immunoprecipitated Using Time Course Infection Sera. Pathogens. 2021, 10, 749.
